# The Punishment of Rape in England and the Colonies

**Published:** 1910-01-01

**Authors:** 


### SPECIAL ARTICLES.

### THE PUNISHMENT OF RAPE IN ENGLAND AND THE COLONIES.

One of the worst cases of rape was tried by Mr. Justice Pickford at Leicester, on October 27. The prisoner, John William Glenn (30), a shoemaker, was indicted for rape upon a hospital nurse at Melton Mowbray, on September 13. The case for the prosecution was that the nurse, while proceeding from the hospital to the station on a dark night, was attacked by the prisoner, who first partially stunned her with blows on the head and then dragged her across a field to a hovel, where he committed the offence. The young lady's screams had attracted attention, and several police officers searched the neighbourhood with lanterns, ultimately finding the prisoner with her in the hovel. The prosecutrix was in a pitiable condition. She was unconscious and suffering from serious injuries. She was conveyed to the hospital, and remained for weeks in a precarious condition. The prisoner, who persisted in denying the assault, was found guilty. His lordship, in sentencing him to 15 years' penal servitude, said that it was the worst case of rape he ever remembered. The details of the prisoner's violence were incomprehensible and almost unnatural.

In early times it was incumbent on the woman who brought an appeal of rape to prove that while the offence was recent she raised " hue and cry " in the neighbouring towns, and showed her injuries and clothing to men, and the appellee might raise as a defence the denial that she had raised the hue and cry. The passages in Bracton (*De Corona* lib. iii, fol. 147), and in the Pleas of the Crown, (Selden Society, vol. i, Warwickshire Eyre, A.D. 1221, p. 109); as also in the Mirror of Justices (Selden Society, vol. vii, Exceptions, cap. xxi, p. 103) are interesting and instructive, as showing that the distinction in this special class of case has been recognised from the earliest times. The hue and cry was the phrase used generally for the pursuit of a felon; but no other case can be found other than rapes in which it was for the prosecution to show that they had raised it, or in which it was a defence to show that it had not been raised. Bracton (De Corona, fol. 104 b) classes rape of a virgin or nun, or of a matron living honestly, with crimes of a somewhat minor character concerning partly the King on account of the breach of his peace, and partly private persons against whom a delict has been committed. Rape has been defined to be the having unlawful and carnal knowledge of a woman by force and against her will. This offence formerly was, for many years, visited with capital punishment; but it does not appear to have been regarded as equally heinous at all periods of our history. Anciently, indeed, it appears to have been treated as a felony, and consequently punishable with death; but this was afterwards thought too hard, and in its stead another severe, but not capital punishment, was inflicted by William the Conqueror, namely, castration and loss of eyes. The punishment for rape was still further mitigated in the reign of Edward I. by the statute of Westm. 1, c. 13, which reduced the offence to a trespass, and subjected the party to two years' imprisonment and a fine at the King's will. This lenity, however, is said to have been productive of terrible consequences; and it was therefore found necessary in about ten years afterwards, and in the same reign, again to make the offence of forcible rape a felony by the statute of Westm. 2, c. 34. By that statute it was enacted that if thereafter a man should ravish a woman, married, maid, or other, where she did not consent neither before or after, he should have judgment of life and member, even if the woman, after being deforced, afterwards consented. From that date the offence was a capital felony. The punishment was still further enhanced by the 18 Eliz. c. 7, s. 1. But the former statutes are repealed. And now by the 24 and 25 Vict. c. 100, s. 48 it is enacted that " Whosoever shall be convicted of the crime of rape shall be guilty of felony, and being convicted thereof shall be liable to be kept in penal servitude for life."

The essence of the offence is that force be used, and it is immaterial what is the age of the woman, and whether she is married or single, chaste or unchaste. The only difference caused by the habitual unchastity of the woman is that in such a case it is less easy to satisfy the jury that the element of consent was wanting. The two elements of rape are the carnal knowledge and the force used. Much difficulty has arisen in defining the meaning of *carnal knowledge*, and different opinions have been entertained—some judges having supposed that penetration alone is sufficient, while others deemed emission an essential ingredient in the crime. It was once held that in order to commit the crime of rape it is requisite that the penetration should be such as to rupture the hymen *Rex* v. *Gammon* (1832, 5 C. and P., p. 321). But this case was subsequently overruled by *R.* v. *Hughes* (1841, 9 C. and P., p. 753). The law of England presumes that an infant under the age of fourteen years is unable to commit the crime of rape, and therefore he cannot be guilty of it, or of an assault with intent to commit a rape; and if he be under that age no evidence is admissible to show that in point of fact he could commit the offence. By the Scotch law there is no restriction as to the age at which a boy may commit rape. A boy under fourteen has been convicted (Fulton 1841, 2 Swin. 564). A charge of rape can be tried only before the Court of Justiciary. The offence was by statute capital (Act 1612, c. 4) until the Criminal Procedure Act, 1887, s. 56. The punishment now usually imposed is penal servitude for a long term, or for life. The offence is now bailable (51 and 52 Vict. c. 36, s. 2).

The American Law.

By the United States Statutes it is enacted that if any persons upon the high seas, or in any arm of the sea, or in any river, haven, creek, basin, or bay, within the Admiralty and maritime jurisdiction of the United States, and out of the jurisdiction of any particular States, shall commit the crime of rape . . . every person so offending, his counsellors, aiders, and abettors, shall be deemed guilty of felony, and shall, upon conviction thereof, suffer death (Act 3rd March, 1825, s. 4). If any person upon the high seas or in any of the other places aforesaid with intent to commit a rape, or do or perpetrate any other felony (shall break or enter any vessel, boat, or raft) . . . shall on conviction thereof be punished by fine, not exceeding one thousand dollars, and by imprisonment and confinement to hard labour not exceeding five years (*Ibid.* s. 7).

By the laws of Kentucky it is provided that "Whosoever shall be guilty of the crime of rape upon the body of an infant under twelve years of age shall be punished with death, or with confinement in the penitentiary for life, in the discretion of the jury" (General Statutes c. 29 a. 4 s. 3). "Whosoever shall unlawfully carnally know a female, of and above twelve years of age, against her will and consent, or by force; or whilst she is insensible, shall be guilty of rape and punished by confinement in the penitentiary not less than ten nor more than twenty years, or by death in the discretion of the jury" (*Ib.* s. 5). "Whosoever shall carnally know a female under the age of twelve years, or an idiot, shall be confined in the penitentiary not less than ten nor more than twenty years" (*Ib.* s. 6).

According to the law prevailing in the State of Indiana, "Whoever unlawfully has carnal knowledge of a woman against her will, or of a female child under fourteen years of age, is guilty of rape, and upon conviction thereof shall be imprisoned in the State prison not more than twenty-one years nor less than one year."

In Kansas the punishment for rape is hard labour for not less than five years nor more than twenty-one years (General Statutes 1868, ch. 31, s. 31, as amended by law 1887, ch. 150, s. 1). By the New York Statutes the crime is punishable with imprisonment for not less than five years nor more than twenty. No conviction for rape can be had against anyone who was under the age of fourteen years at the time of the act alleged, unless his physical ability to accomplish penetration is proved as an independent fact, beyond a reasonable doubt. Any sexual penetration, however slight, is sufficient to complete the crime. A person who, under promise of marriage, seduces and has sexual intercourse with an unmarried female of previous chaste character, is punishable by imprisonment for not more than five years, or by a fine of not more than one thousand dollars, or both (Penal Code ss. 278—286).

There is a recent trend in legislation in America in the direction of raising the age of consent. This has resulted from a very active agitation on the subject largely promoted by the societies for the prevention of cruelty to children and persons who devote themselves especially to the promotion of social purity. By recent legislation the age of consent is made in Virginia, Wisconsin, and Indiana, fourteen years; in Iowa, Texas, and Delaware, fifteen years; Connecticut, Louisiana, Massachusetts, Michigan, Oregon, and South Dakota, sixteen years; and in Arizona, Colorado, Idaho, Missouri, Nebraska, New York, Utah, and Wyoming, it is eighteen years.

The Australian Colonies.

By the Crimes Act, 1900 (No. 40), of New South Wales, s. 63, it is provided that "Whosoever commits the crime of rape shall be liable to suffer death. The consent of the woman, if obtained by threats or terror, shall be no defence to a charge under this section." Section 67 enacts that "Whosoever carnally knows any girl under the age of ten years shall be liable to suffer death."

In Victoria the Crimes Act, 1890 (No. 1079), enacts by s. 42 that "Whosoever shall be convicted of the crime of rape shall be guilty of felony and suffer death." By s. 46, "Whosoever shall unlawfully and carnally know and abuse any girl under the age of ten years shall be guilty of felony, and, being convicted thereof, shall suffer death."

By the criminal law of Tasmania rape was formerly punishable with death, but by an Act passed in 1885 the punishment has been altered to penal servitude for life.

By s. 299 of the Criminal Code of Canada "everyone who commits rape is guilty of an indictable offence and liable to suffer death or to imprisonment for life."

